# The Effects of SGLT2 Inhibitors on Lipid Metabolism

**DOI:** 10.3390/metabo11020087

**Published:** 2021-02-01

**Authors:** Zsolt Szekeres, Kalman Toth, Eszter Szabados

**Affiliations:** 1 1st Department of Medicine, Division of Preventive Cardiology and Rehabilitation, University of Pecs, Medical School, H-7624 Pecs, Hungary; szekeres.zsolt@pte.hu; 2 1st Department of Medicine, Division of Cardiology, University of Pecs, Medical School, H-7624 Pecs, Hungary; toth.kalman@pte.hu

**Keywords:** SGLT2 inhibitors, type 2 diabetes mellitus, lipid metabolism

## Abstract

Sodium glucose co-transporter 2 (SGLT2) inhibitors are effective antihyperglycemic agents by inhibiting glucose reabsorption in the proximal tubule of the kidney. Besides improving glycemic control in patients with type 2 diabetes, they also have additional favorable effects, such as lowering body weight and body fat. Several clinical studies have demonstrated their positive effect in reducing cardiovascular morbidity and mortality. Furthermore, the use of SGLT2 inhibitors were associated with fewer adverse renal outcomes comparing to other diabetic agents, substantiating their renoprotective effect in diabetic patients. SGLT2 inhibitors have also remarkable effect on lipid metabolism acting at different cellular levels. By decreasing the lipid accumulation, visceral and subcutaneous fat, they do not only decrease the body weight but also change body composition. They also regulate key molecules in lipid synthesis and transportation, and they affect the oxidation of fatty acids. Notably, they shift substrate utilization from carbohydrates to lipids and ketone bodies. In this review we intended to summarize the role of SGLT2 inhibitors in lipid metabolism especially on lipoprotein levels, lipid regulation, fat storage and substrate utilization.

## 1. Introduction

**Figure 1:**
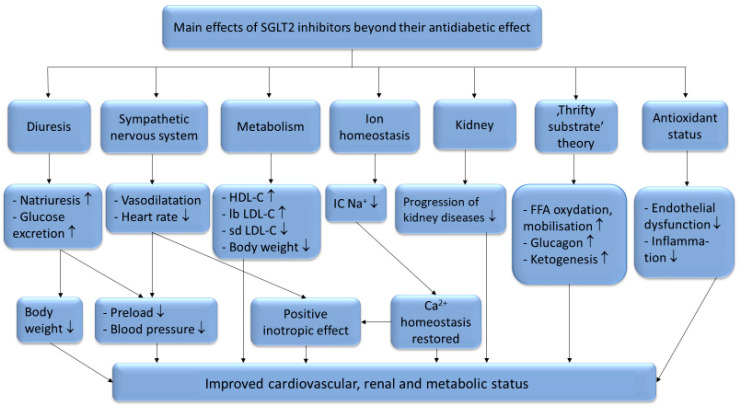
The systematic effects of SGLT2-inhibitors. HDL-C: high-density lipoprotein cholesterol; lb LDL-C: large buoyant low-density lipoprotein cholesterol; sd LDL-C: small dense low-density lipoprotein cholesterol; TG: triglycerides; IC Na^+^: intracellular sodium-ion; FFA: free fatty acids; ↑: increased amount; ↓: decreased amount.

There are six identified SGLT (sodium glucose co-transporter) proteins in humans, of which the SGLT1 and SGLT2 receptors have been studied more thoroughly in recent years. Despite the outstanding sequence similarity between SGLT1 and SGLT2, they show different physiological and biochemical properties. While SGLT1 is primarily expressed in the intestines, SGLT2 is most abundant in the renal cortex, where it plays an essential role in renal glucose reabsorption. SGLT2 inhibitors, including dapagliflozin, canagliflozin, ipragliflozin, tofogliflozin, luseogliflozin, sotagliflozin, ertugliflozin and empagliflozin, have been studied in several clinical studies for the treatment of type 2 diabetes mellitus. Their selectivity for the SGLT2 receptor shows significant variance. While empagliflozin is 2500×, ertugliflozin is 2000×, dapagliflozin is 1200×, canagliflozin is 250×, sotagliflozin is only 20× more selective for the SGLT2 receptor over SGLT1, which may cause significant differences in the mechanism of action [1]. Several studies have proved that SGLT2 inhibitors are associated with reduced cardiovascular morbidity and mortality, including heart failure, and vascular diseases [2,3,4,5,6]. The underlying hypothetic mechanisms of SGLT2 inhibitors beyond their antidiabetic effects are summarized in Figure 1.

Furthermore, SGLT2 inhibitors have renoprotective effects as well [7]. Besides their beneficial effects on the conventional risk factors for kidney disease (such as blood pressure, hyperglycemia, body weight), it has also been hypothesized that they reduce the intraglomerular pressure [8], change the activation of the renin-aldosterone-angiotensin system [9] and shift renal fuel consumption towards ketone bodies [10].

Interestingly, these cardiovascular and renoprotective effects occur despite an increase in low-density lipoprotein cholesterol (LDL-C) levels which was observed in several clinical studies [11,12]. Nevertheless, this increase in LDL-C happens in the setting of other beneficial changes in plasma lipoprotein metabolism. In this review, we aim to analyze the effects of SGLT2 inhibitors on lipid metabolism to better understand their beneficial effects on cardiovascular outcomes despite slightly elevating LDL-C level.

## 2. Methods

A systematic literature search was conducted to identify all studies that investigated the SGLT2 inhibitor therapy on lipid metabolism in the PubMed (pubmed.ncbi.nlm.nih.gov) database. The searched terms were: SGLT2 inhibitors and metabolic effects, lipoprotein levels, lipid metabolism, lipid regulation and lipid accumulation. Only original research articles, review articles and meta-analyses published in the English language were selected.

## 3. The SGLT2 Inhibitors’ Effect on Plasma Lipoprotein Levels

Several studies reported lowered serum levels of total cholesterol (TC) and triglycerides (TG) [13,14,15] as a result of SGLT2 inhibitor therapy, however there is a debate regarding the changes observed in the serum levels of high-density lipoprotein cholesterol (HDL-C) and low-density lipoprotein cholesterol (LDL-C). According to Calapkulu et al. LDL cholesterol level decreased by 13.4 mg/dL after 6 months in diabetic patients with dapagliflozin (10 mg/die) [13], In contrast, Cha et al. reported an increase of 1.3 mg/dL in LDL level after 24 weeks of dapagliflozin add-on therapy [16], in concordance with the results of Basu et al. in mice [17]. Furthermore, according to Schernthaner et al. canagliflozin (300 mg/die) caused 11.7% increase in LDL after 52 weeks of therapy in patients with type 2 diabetes mellitus [18]. According to Basu et al. the cause of this possible increase in the LDL-C levels could be due to an increased lipoprotein-lipase (LpL) activity and because of a delayed turnover of LDL in the circulation. Canagliflozin reduced the expression of angiopoetin-like protein 4 (ANGPTL4), which is a known inhibitor of LpL in white and brown adipose, skeletal muscle, and heart tissues. With greater LpL activity both the TG and the VLDL levels decreased. They also observed significantly delayed LDL turnover compared to the control group, which can originate from the lowered hepatic levels of the LDL-receptor, which is the major receptor for the clearance of plasma LDL [17]. Another important factor is the ratio of the different LDL subclasses. Using gradient gel electrophoresis (GGE) LDL particles are classified into 4 subclasses, including large (LDL I), intermediate (LDL II), small (LDL III), and very small (LDL IV) LDLs [19,20]. LDL I and II, also referred to as large buoyant (lb) LDL, and LDL III and IV as small dense (sd) LDL particles [21,22]. Small dense LDL particles are more prone to induce metabolic disorders [23,24], obesity [25,26], type 2 diabetes [20,27] and coronary artery disease (CAD) [28] due to their longer circulation time than that of large LDL particles [29], enhanced ability to penetrate the arterial wall and higher susceptibility to oxidation [30]. It is generally known that modified (oxidized and glycated) LDL particles are highly atherogenic and possess more proinflammatory properties than native LDL molecules [28,31]. Interestingly SGLT2 inhibitors slightly increase LDL level yet have beneficial effects on CV morbidity and mortality. The contradiction may be resolved by the results provided by Hayashi et al. showing that dapagliflozin decreased sd LDL, and increased lb LDL levels after 12 weeks of dapagliflozin therapy (5 mg/die) in type 2 diabetic patients [15]. This effect on LDL subclasses ratio may play a significant role in SGLT2 inhibitors’ cardioprotective property [32,33], provided it is a class effect.

Concerning HDL level, according to Kamijo et al. after 12 weeks of canagliflozin administration (100 mg/die) the very large high-density lipoprotein (VLHDL) and large high-density lipoprotein (LHDL) values showed a significant increase, of 10.9% and 11.5% respectively. These beneficial changes might also contribute to subsequent reduction of cardiovascular outcomes, caused by SGLT2 inhibitors [34].

## 4. The SGLT2 Inhibitors’ Effect on Lipid Regulation

Several signaling molecules have been measured in mice after a 4-week treatment with canagliflozin by Ji et al. [14]. Elevated levels of diacylglycerol O-acyltransferase 2 (DGAT2) mRNA were reversed by canagliflozin. They also observed an increase in peroxisome proliferator-activated receptor-α (PPAR-α), and a decrease in peroxisome proliferator-activated receptor-γ (PPAR-γ) levels [14]. DGAT2 is an integral membrane protein which promotes the synthesis and storage of TG in lipid droplets. The peroxisome proliferator-activated receptors (PPAR) are a group of nuclear receptor proteins that function as transcription factors regulating the expression of several genes. Three types of PPARs have been identified: alpha (α), gamma (γ), and beta/delta (β/δ). While the PPAR-α is expressed mostly in the liver, kidneys, heart, muscle and adipose tissue, it mainly regulates the lipid metabolism in the liver. It is activated under conditions of energy deprivation, and it is necessary for the process of ketogenesis. Activation of PPAR-α promotes the uptake, utilization and catabolism of fatty acids through upregulating the genes that are involved in fatty acid binding and activation, peroxisomal and mitochondrial fatty acid β-oxidation. PPAR-γ regulates the fatty acid storage and glucose metabolism. It activates genes stimulating lipid uptake and adipogenesis in fat cells, it also plays a crucial role in adipocyte differentiation. PPAR-γ increases insulin sensitivity through increasing storage of fatty acids in fat cells, enhancing adiponectin release, inducing fibroblast growth factor 21 (FGF21) and upregulating the cluster of differentiation 36 (CD36) enzyme [35]. This data indicates that canagliflozin suppressed the synthesis of TG and the accumulation of hepatic lipid droplets through the down regulation of DGAT2.

Mice treated with canagliflozin had significant increase in both hepatic and serum FGF21 levels [36]. FGF21 is a fasting-induced hepatokine that stimulates glucose uptake in adipocytes, but not in other cell types. FGF21 acts through the Ras/MAP kinase pathway. This indicates, that canagliflozin triggered a fasting-like catabolic switch, increasing the adipose lipolysis, hepatic fatty acid oxidation and ketogenesis, potentially via FGF21-dependent mechanisms. In addition, FGF21 can induce sympathetic activation in the central nervous system, leading to energy expenditure and weight loss. According to Osataphan et al. FGF21 was essential for the SGLT2 inhibitor induced reduction in adipose tissue mass, adipocyte cell size and activation of lipolysis. While canagliflozin reduced adipocyte size, FGF21-null mice had no weight loss, and adipose tissue and cell size even increased in response to canagliflozin, suggesting that FGF21-null mice had impaired sympathetic and lipolytic activity. FGF21 is also responsible for increasing oxidative metabolism, browning and lipolysis in white adipose tissue [36].

According to the findings of Herrera et al., empagliflozin therapy significantly reduced the gene expression as well as the protein levels of CD36 in atrial tissues of rats after 6 weeks [37]. CD36, also known as fatty acid translocase (FAT), is an integral membrane protein found on the surface of cells that import fatty acids inside cells. It is also a member of the class B scavenger receptor family. CD36 interacts with several ligands, including collagen types I and IV, oxidized LDL and long-chain fatty acids as well. It is also involved in the macrophages’ phagocytosis. After CD36 binds to a ligand, they are internalized thus long-chained fatty acids and oxidized LDL particles can enter the cells. Since autophagy is decreased in metabolic disorders like diabetes and obesity, this dysregulation is an important factor in the pathophysiology of heart failure. The results show, that empagliflozin may ameliorate the impaired basal cardiac levels of autophagy that would lead to the aggregation of proteins, at least in part through CD36, which contributes to the pathogenesis of cardiometabolic diseases.

Xu et al. showed, that empagliflozin elevated AMPK and ACC-CoA phosphorylation in skeletal muscle, and increased hepatic and plasma FGF-21 levels. Empagliflozin also increased energy expenditure, heat production, and the expression of uncoupling protein 1 in brown fat and in inguinal and epididymal white adipose tissue. The M1-polarized macrophage accumulation was reduced, while plasma TNFα levels and obesity-related chronic inflammation decreased. In summary, empagliflozin did not just suppress weight gain by inducing fat utilization and browning, but also attenuated obesity-induced inflammation and insulin resistance [38]. Likewise, Osataphan et al. concluded that canagliflozin therapy activated AMPK through the inhibition of mitochondrial complex I, without an increase in ACC-CoA. However, this change in AMPK phosphorylation was not present in lean mice, thus AMPK is not likely to be the major mediator for the increase in fatty acid oxidation and ketogenesis [36]. One of the key molecules in the transport and oxidation of fatty acids is acetyl-CoA carboxylase (ACC), which converts acetyl-CoA to malonyl-CoA. Malonyl-CoA is an inhibitor of carnitine palmitoyltransferase 1 (CPT-1), which transports fatty acids into the mitochondria for oxidation. Thus, inactivation of ACC results in increased fatty acid transport and subsequent oxidation. On the other hand, AMP-activated protein kinase (AMPK) may decrease malonyl-CoA levels by regulating malonyl-CoA decarboxylase (MCD). Another important role of AMPK is, that it phosphorylates and inactivates 3-hydroxy-3-methylglutaryl-CoA reductase (MHGCR), which is a key enzyme in cholesterol synthesis. AMPK, therefore, regulates fatty acid oxidation and cholesterol synthesis [38]. Mammalian target of rapamycin (mTOR) is a cellular energetic sensor, which is often regulated inversely with AMPK. Osataphan et al. found that after canagliflozin therapy there was a 56% decrease in the hepatic phosphorylation of the mTOR downstream substrate S6 when refeeding in canagliflozin-treated mice. This change was not present in the control groups, thus the decrease in mTOR signaling might be weight-dependent [36].

Empagliflozin therapy reduced cardiac content of sphingolipids (sphingomyelins and ceramides) and glycerophospholipids, which play an important role in connecting insulin resistance to cardiac damage, and even in the development of cardiovascular diseases. It is suggested that changes in lipid metabolism within the heart and cardiac lipid accumulation may have an important role in the development of diabetic cardiomyopathy and heart failure. Ceramides, sphingomyelins and glycerophospholipids are associated with lipotoxicity in the heart, thus they have a major impact on the organ’s functionality. This suggests, that empagliflozin could regulate the metabolism and the cardiac accumulation of these cardiotoxic lipid molecules, which means, that it could be potentially useful for the prevention and treatment of not only diabetic cardiomyopathy, but also for the management of other cardiovascular diseases that have lipotoxicity in their pathogenesis [37].

## 5. The SGLT2 Inhibitors’ Effect on Metabolism

Chiang et al. investigated a novel SGLT2 inhibitor’s, NGI001, effect on non-alcoholic fatty liver disease (NAFLD) and obesity-associated metabolic symptoms in high-fat diet-induced obese mice. According to their results NGI001 prevented adipocyte hypertrophy, inhibited impaired glucose metabolism and regulated the secretion of adipokines associated with insulin resistance. Notably, NGI001 suppressed hepatic lipid accumulation and inflammation. NGI001 ameliorated fat deposition and increased AMPK-phosphorylation, resulting in ACC-CoA phosphorylation. In addition, it blocked the storage of total fat in adipose tissue and alleviated TG accumulation in liver tissue, and the organ’s weight decreased likewise. Interestingly, they found that the TG and cholesterol level in the faeces increased. This effect on the lipid excretion through the intestinal system needs further study [39]. Yokono et al. investigated the effects on ipragliflozin on body weight and composition in mice. They found that 4 weeks of therapy suppressed body weight increase despite the small increase in food intake. The reduction of body weight was accompanied by reduced visceral and subcutaneous fat masses. Ipragliflozin lowered the respiratory exchange ratio and decreased the heat production rate from glucose but increased it from fat, thus ipragliflozin mainly promoted the use of fatty acids instead of glucose as an energy source [40]. Other studies showed similar results with different SGLT2 inhibitors, like dapagliflozin [41,42], canagliflozin [43], and empagliflozin [44]. In summary, these results suggest that the weight loss during SGLT2 inhibitor therapy may result from the reduction in fat tissue content via enhanced fatty acid utilization.

**Figure 2:**
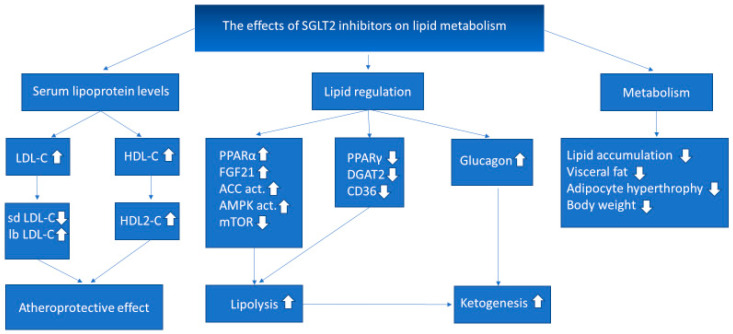
The effects of SGLT2 inhibitor therapy on lipid metabolism. LDL-C: low density lipoprotein cholesterol; sd LDL-C: small dense low-density lipoprotein cholesterol; lb LDL-C: large buoyant low-density lipoprotein cholesterol; HDL-C: high-density lipoprotein cholesterol; PPARα: peroxisome proliferator-activated receptor α; FGF21: fibroblast growth factor 21; ACC act.: acetyl-CoA carboxylase activation; AMPK act.: AMP-activated protein kinase activation; mTOR: mammalian target of rapamycin; PPARγ: peroxisome proliferator-activated receptor γ; DGAT2: diacylglycerol O-acyltransferase 2; CD36: cluster of differentiation 36; ↑: increased amount; ↓: decreased amount.

The SGLT2 inhibitor therapy increases the production of ketone bodies through various pathways. The decreased level of glucose in the blood increases the production of glucagon, which promotes ketogenesis. Also, SGLT2 receptor was found on the surface of pancreatic α-cells that can act as a glucose sensor [45]. On the other hand, the increased lipolysis also promotes the production of ketone bodies in the liver [46]. The oxidation of the ketone bodies is energetically more efficient than the oxidation of fatty acids because it results in a higher ATP/oxygen ratio than other substrates. According to the ‘thrifty substrate’ theory under conditions of mild, persistent hyperketonemia, such as during SGLT2 inhibitor therapy, β-hydroxybutyrate is freely taken up by the heart and oxidized instead of fatty acids and glucose [47]. Taking into consideration that ketone bodies serve as an alternative and less expensive myocardial fuel source to the myocardium SGLT2 inhibitors may improve cardiac function and increase mechanical efficiency [48,49]. Furthermore, beta-hydroxybutyrate has antioxidant and antiarrhythmic effects, by inhibiting histone deacetylases, by upregulating mitochondrial biogenesis and, by stabilizing cell membrane potential [47]. Metabolic flexibility means the ability of muscle to switch between FFAs and glucose as the main fuel source based on substrate availability. Patients with diabetes have impaired metabolic flexibility, thus the myocardium becomes more reliant on FFA oxidation. This impairment is the result of the increased FFA delivery to the heart due to peripheral insulin resistance and due to insulin’s inability of suppressing lipolysis. This causes increased myocardial FFA oxidation and reduced glucose oxidation. FFAs also impair insulin action by inhibiting insulin signaling pathways, what leads to decreased cellular glucose transport, and even less glucose oxidation. This increase in FFA oxidation decreases cardiac efficiency and cause lipotoxicity. The hyperglycemia causes glucotoxicity, which is associated with reactive oxygen species (ROS) overproduction, which has a negative effect on mitochondrial function and other cellular processes. Ketone bodies are alternative fuel for the cells, that played a critical role in human survival. They are almost exclusively synthesized in the liver in the case of high circulating FFA levels, and when the production of ACC-CoA exceeds hepatic cellular energy requirements. The ketone bodies then diffuse into the circulation and into extrahepatic tissues, mainly the heart and kidney, providing a major energy source during fasting [10]. It is documented that ketone bodies are mildly elevated when SGLT2 inhibitors are administered to patients [50]. The myocardium is the highest consumer of ketone bodies per unit mass, and it is shown, that despite impairments in skeletal muscle ketone body utilization, myocardial ketone body utilization was preserved in heart failure [51]. However, we must also consider, that euglycemic diabetic ketoacidosis (DKA) may be a rare, but severe side effect of SGLT2 inhibitor drugs, observed mostly under conditions favorable to excessive ketogenesis, such as increased alcohol consumption, volume loss, infection, stroke, and myocardial infarction [52]. In summary, SGLT2 inhibition in normal conditions through substrate shift offers the myocardium an alternative fuel that increases cardiac efficiency and decreases lipotoxicity. The main effects of SGLT2 inhibitors on lipid metabolism are summarized in Figure 2.

## 6. Conclusions

SGLT2 inhibitors affect the lipid metabolism on several different levels. They decrease lipid accumulation in visceral fat, regulate the serum lipoprotein levels, beneficially change the ratio of LDL particles, reduce lipid oxidation, and shift substrate utilization towards the usage of ketone bodies, which are more efficient in myocardial metabolism, and less reactive oxygen species are created through their oxidation, affect the β-oxidation and the transportation of lipid molecules in the cells. These favorable changes in lipid metabolism may counteract the net increase in LDL level. These findings may show that even though SGLT2 inhibitors are used primarily for the treatment of patients with type 2 diabetes, they may not be restricted merely to these indications in the near future.

## 7. Strengths and Limitations

Numerous relevant clinical and basic research studies and reviews have been included in this review article to summarize the potential effects of SGLT2 inhibitors on lipid metabolism giving a better understanding about the complex molecular mechanisms offered by these agents.

The limitations of this study are that we have described only the most important molecular mechanisms and searched only PubMed database for English language articles.
